# Fertility of CMS wheat is restored by two *Rf* loci located on a recombined acrocentric chromosome

**DOI:** 10.1093/jxb/eru388

**Published:** 2014-09-30

**Authors:** Almudena Castillo, Sergio G. Atienza, Azahara C. Martín

**Affiliations:** ^1^Instituto de Agricultura Sostenible, IAS-CSIC, Apdo. 4084, Córdoba E-14080, Spain; * Present address: John Innes Centre, Norwich Research Park, Norwich NR4 7UH, United Kingdom

**Keywords:** Acrocentric chromosome, cytoplasmic male sterility, Hordeum chilense, restorer gene, Triticum aestivum, zebra-like chromosome.

## Abstract

Cytoplasmic male sterility (CMS) results from incompatibility between nuclear and cytoplasmic genomes, and is characterized by the inability to produce viable pollen. The restoration of male fertility generally involves the introgression of nuclear genes, termed restorers of fertility (*Rf*). CMS has been widely used for hybrid seed production in many crops but not in wheat, partly owing to the complex genetics of fertility restoration. In this study, an acrocentric chromosome that restores pollen fertility of CMS wheat in *Hordeum chilense* cytoplasm (msH1 system) is studied. The results show that this chromosome, of *H. chilense* origin and named H^ch^ac, originated from a complex reorganization of the short arm of chromosomes 1H^ch^ (1H^ch^S) and 6H^ch^ (6H^ch^S). Diversity arrays technology (DArT) markers and cytological analysis indicate that H^ch^ac is a kind of `zebra-like′ chromosome composed of chromosome 1H^ch^S and alternate fragments of interstitial and distal regions of chromosome 6H^ch^S. PCR-based markers together with FISH, GISH, and meiotic pairing analysis support this result. A restorer of fertility gene, named *Rf*_*6H*_^*ch*^_*S*_, has been identified on the short arm of chromosome 6H^ch^S. Moreover, restoration by the addition of chromosome 1H^ch^S has been observed at a very low frequency and under certain environmental conditions. Therefore, the results indicate the presence of two *Rf* genes on the acrocentric chromosome: *Rf*_*6H*_^*ch*^_*S*_ and *Rf*_*1H*_^*ch*^_*S*_, the restoration potential of *Rf*_*6H*_^*ch*^_*S*_ being greater. The stable and high restoration of pollen fertility in the msH1 system is therefore the result of the interaction between these two restorer genes.

## Summary

The high potential for an acrocentric chromosome originated from a complex reorganization of chromosomes 1H^ch^S and 6H^ch^S from *Hordeum chilense* in the development of hybrid wheat technology.

## Introduction

Global demand and consumption of agricultural crops for food, animal feed, and fuel are increasing at a rapid rate. Current projections indicate that the world population could increase by 2.25 billion people from present numbers, reaching 9.15 billion by 2050. To feed this larger, increasingly affluent population, food production must increase by 70 percent, and annual cereal production will need to rise from the present 2.8 tonnes ha^–1^ to 3.8 by 2050 (Alexandratos and Bruinsma, 2012). Wheat, as one of the most consumed cereals, together with rice and maize, is confronting a new challenge. Furthermore, climate change threatens the world’s wheat crop production. This means that productivity needs to be improved, and new varieties adapted to the changing situation must be obtained to produce the additional 200 million tonnes per year already estimated to be needed by 2017 (Edgerton, 2009).

Heterosis, associated with the superiority of the first filial generation over the parental generation, is a powerful tool for improving yield and quality in many crops. The main advantages of hybrid versus line varieties are increased trait values owing to the exploitation of heterosis (Shull, 1948), greater yield stability especially in marginal environments (Hallauer *et al.*, 2010; Mühleisen *et al.*, 2014), and the ease of stacking dominant major genes (Edwards, 2001). Hybrid technology has been successfully exploited in several crops such as rice, maize, sunflower, sorghum, sugar beet, and rye (Hallauer, 1999). However, despite great efforts invested in the development of commercially viable hybrid wheat technology, to date, less than 1% of the total world wheat area is planted with hybrids (Longin *et al.*, 2012). The reasons for this are diverse and include: the autogamous nature of wheat, its hexaploid condition, and the well-established use of line varieties. Ultimately, the real issue is the lack of an optimum system for hybrid production. In 2008, a new cytoplasmic male sterility (CMS) source in bread wheat (Triticum aestivum), designated msH1, was described (Martín *et al.*, 2008*a*). This system uses the cytoplasm of *Hordeum chilense* Roem. *et* Schult. accession H1 (2n=2x=14, H^ch^H^ch^), a diploid wild barley native to Chile and Argentina, which possesses some useful traits for wheat breeding such as drought and salt tolerance, resistance to several pests and diseases (Martín *et al.*, 1996), high seed carotenoid content (Atienza *et al*., 2004, 2007), and high crossability with other members of the Triticeae tribe: *Aegilops*, *Agropyrum*, *Dasypyrum*, *Secale*, *Triticum*, and ×*Triticosecale* (Bothmer and Jacobsen, 1986; Martín *et al.*, 1998). The msH1 CMS source in bread wheat is stable under varying environmental conditions, and neither grain shrivelling nor germination disorders have been observed. Unlike many other alloplasmic genotypes of wheat that exhibit developmental or floral abnormalities, the msH1 system shows only slightly reduced height and 3–4 days delay in heading (Martín *et al.*, 2008*a*). Fertility restoration of the CMS phenotype was associated with the addition of the short arm of chromosome 6H^ch^ of *H. chilense* (Martín *et al.*, 2008*a*). When the alloplasmic ditelosomic addition line of 6H^ch^S was obtained, it was fully fertile; however, a single dose of 6H^ch^S was not sufficient for fertility restoration. Therefore, different wheat varieties and different chromosome combinations were explored in the search for new restorer genes. In 2010, whilst testing the msH1 system in different wheat backgrounds, a highly fertile line with 42 wheat chromosomes plus an extra acrocentric chromosome was obtained (Martín *et al.*, 2010). The novel chromosome, named H^ch^ac, was able to restore fertility even in monosomic condition, which made it a good candidate for use in a hybrid production system. Data obtained from FISH (fluorescence i*n situ* hybridization) and EST (expressed sequence tag) markers suggested that the long arm of the H^ch^ac chromosome was the short arm of chromosome 1H^ch^ from *H. chilense*. The hypothesis was that the novel chromosome originated from chromosome 1H^ch^ after a deletion of the distal part of the long arm of 1H^ch^ (1H^ch^L). As neither the 1H^ch^S arm, nor the whole chromosome 1H^ch^ restored pollen fertility of the alloplasmic wheat, it was hypothesized that the restorer gene on the acrocentric chromosome was located on the retained segment from chromosome 1H^ch^L, whereas some pollen fertility inhibitor was present on the deleted 1H^ch^L distal segment. However, the door was open to a more complicated origin of the acrocentric chromosome.

In the present work we continue the previous study to clarify the nature of the *H. chilense* chromosomes involved in the formation of H^ch^ac, as well as its role in the restoration of pollen fertility in the msH1 system. As it was shown that the whole H^ch^ac was of *H. chilense* origin, the use of GISH (genomic i*n situ* hybridization) was not suitable. Instead, DArT (diversity arrays technology) molecular markers were used to clarify the situation, and found out that indeed, the extra acrocentric chromosome was produced by a more complicated process than that originally described. We demonstrate that H^ch^ac is a zebra-like chromosome (Jiang and Gill, 1993; Zhang *et al.*, 2008) originating from chromosomes 6H^ch^S and 1H^ch^S. We compared restoration capability of the addition of 6H^ch^S or 1H^ch^S chromosome arms in the alloplasmic wheat, with that of the 1HS^ch^+6H^ch^S addition, and found that stable and high restoration of pollen fertility is obtained by the combination 1H^ch^S+6H^ch^S. Therefore we propose the presence of two restorers of fertility genes (*Rf*_*6H*_^*ch*^_*S*_ and *Rf*_*1H*_^*ch*^_*S*_) in the H^ch^ac chromosome.

## Material and methods

### Plant material

The genetic stocks used in this work are detailed in Table 1. *T. aestivum* cv. Chinese Spring (CS)-*H. chilense* addition lines (T21A1H_1_S, T21A1H_1_-1H1S, and T21A6H_1_S) were kindly provided by Steve Reader, JIC, Norwich, UK. Lines T218 and T593 were described in Martín *et al*. (2008*a*). Lines T236, T526, and T528 were developed by Martín *et al*. (2010). Lines T700 and T749 were obtained in this work. CS-*H. chilense* addition lines were used to assign markers to specific chromosomes in the DArT array.

**Table 1.** Description of the plant material used in this studyThe acrocentric chromosome is abbreviated as H^ch^ac.

Line^*a*^	Standard abbreviation^b^	Germplasm^*b*^	Chromosome number^*b*^	Chromosome configuration^*b*^	Fertility
H1	H1	*H. chilense* Roem. et Schultz. accession H1	14	7′′	Fertile
T21	CS	*T. aestivum* cv. Chinese Spring	42	21′′	Fertile
T26	T26	*T. aestivum* cv. T26	42	21′′	Fertile
T236	(H1)T26	*T. aestivum* cv. T26 in H1 cytoplasm	42	21′′	Male sterile
T218	(H1)CS	*T. aestivum* cv. CS in H1 cytoplasm	42	21′′	Male sterile
T526	(H1)T26-H^ch^ MAH^ch^ac	*T. aestivum* cv. T26–*H. chilense* monosomic	42+ac′	20′′ + 1′′ T1RS·1BL +1′ H^ch^ac	Fertile
		addition acrocentric chromosome in H1 cytoplasm			
T528	(H1)T26-H^ch^ DAH^ch^ac	*T. aestivum* cv. T26–*H. chilense* disomic	42+ac′′	20′′ + 1′′ T1RS·1BL +1′′ H^ch^ac	Fertile
		addition acrocentric chromosome in H1 cytoplasm			
T700	(H1)CS-H^ch^ MAH^ch^ac	*T. aestivum* cv. CS –*H. chilense* monosomic	42+ac′	21′′ + 1′ H^ch^ac	Fertile
		addition acrocentric chromosome in H1 cytoplasm			
T749	(H1)CS-H^ch^ DAH^ch^ac	*T. aestivum* cv. CS –*H. chilense* disomic	42+ac′′	21′′ + 1′′ H^ch^ac	Fertile
		addition acrocentric chromosome in H1 cytoplasm			
T21A1H_1_S	CS-H^ch^ DtA1H^ch^S	*T. aestivum* cv. CS–*H. chilense*	42+t′′	21′′ + t′′1H^ch^S	Fertile
		ditelosomic addition 1H^ch^S			
T21A1H_1_-1H_1_S	CS-H^ch^ MA1H^ch^MtA1H^ch^S	*T. aestivum* cv. CS–*H. chilense* monosomic	43+t′	21′′ + 1′ 1H^ch^ + t 1H^ch^S	Fertile
		addition 1H^ch^ monotelosomic addition 1H^ch^S			
T21A6H_1_S	CS-H^ch^ DtA6H^ch^S	*T. aestivum* cv. CS–*H. chilense*	42+t′′	21′′ + t′′6H^ch^S	Fertile
		ditelosomic addition 6H^ch^S			
T593	(H1)CS-H^ch^ DtA6H^ch^S	*T. aestivum* cv. CS–*H. chilense* ditelosomic	42+t′′	21′′ + t′′6H^ch^S	Fertile
		addition 6H^ch^S in H1 cytoplasm			

^*a*^ Abbreviation used in this work

^*b*^ Nomenclature suggested by Raupp *et al.* (1995) for the genetic stocks of wheat and its relatives

### Development of different lines

Lines T700 and T749 were obtained by recurrent back-crossing of T528 to CS. Three backcrosses were sufficient to obtain the CS background in the absence of the 1RS·1BL translocation present in T528. Plants with a single acrocentric chromosome H^ch^ac and with two acrocentric chromosomes were recovered from these crosses and named T700 (42+ac′) and T749 (42+ac′′), respectively. These plants were male fertile.

### Cytological observations

For somatic chromosome counting, root tips of 1-cm length were collected from germinating seeds and pre-treated for 4h in an aqueous colchicine solution (0.05%) at 25 °C. They were fixed in freshly prepared 3:1 of absolute ethanol:glacial acetic acid (*v*/*v*) and stained by the conventional Feulgen technique.

For meiotic chromosome observations, florets were collected and fixed in 3:1 of absolute alcohol:glacial acetic acid (*v*/*v*). The material was transferred to fresh fixative after 1–2h and stored at 4 °C. Anthers were stained with 0.1% acetocarmine.

### Fluorescence *in situ* hybridization (FISH)

Root tips and anthers were fixed as described in “Cytological observations”. Preparations were made as described by Prieto *et al.* (2001).

For GISH, total *H. chilense* genomic DNA was labelled by nick translation with biotin-11-dUTP (Roche Corporation, Basel, Switzerland). Telomere repeat sequence (TRS) probes were labelled with digoxigenin-16-dUTP (Roche Corporation) by nick translation of PCR-amplified products using the oligomer primers (5′-TTTAGGG-3′) and (5′-CCCTAAA-3′) in the absence of template DNA (Cox *et al.*, 1993). The repetitive DNA probe pAs1 (Rayburn and Gill, 1986), isolated from *Aegilops tauschii*, consists of an insert of 185bp (118bp of which constitute the repetitive sequence) in the pGEM-T Easy Vector (Promega, Madison, Wisconsin, USA). Competent cells of *Escherichia coli* (DH5α) were transformed with a plasmid containing the pAs1 probe, and the plasmid was isolated using Plasmid Mini Kit (QIAGEN, Valencia, California, USA). The probe was labelled with digoxigenin-16-dUTP by nick translation. The i*n situ* hybridization protocol was according to that of Cabrera *et al.* (2002). Digoxigenin- and biotin-labelled probes were detected with antidigoxigenin-FITC (Roche Corporate) and streptavidin–Cy3 conjugates (Sigma, St Louis, MO, USA), respectively. Chromosomes were counterstained with DAPI (4′,6-diamidino-2-phenylindole dihydrochloride) and mounted in Vectashield (Vector Laboratories Inc., Burlingame, California, USA). Slides were examined using a Zeiss LSM 5 Pa confocal laser scanning microscope with LSM 5 Pa software version 3.0 (Zeiss, Jena, Germany).

### Molecular analysis

Two replicates of T236, T218, T528, and T700 were analysed. CS- *H. chilense* addition lines were used to assign markers to specific chromosomes. CS, *T. aestivum* cv. T26, and *H. chilense* accession H1 were also included in the analysis. DNA was extracted from young leaf tissue from a single plant of each genotype using the protocol recommended by Triticarte Pty. Ltd., ACT, Australia (http://www.triticarte.com.au). The DNA samples were sent to Triticarte Pty. Ltd. (www.diversityarrays.com) and hybridized to the same resulting composite array which was used to fingerprint tritordeum and wheat-*H. chilense* chromosome addition lines (Castillo *et al.*, 2013). The array consisted mostly of previously developed *H. chilense* and wheat clones, and was completed with markers from barley, rye, and triticale. The resulting composite array was used to genotype alloplasmic and restored lines included in this study using the standard DArT protocol (Kilian *et al.*, 2012). Also wheat- *H. chilense* addition lines were included which enable the assignment of the markers to *H. chilense* chromosomes.

The consensus chloroplast simple sequence repeat ccSSR-4 developed by Chung and Staub (2003) was used to verify the presence of the *H. chilense* cytoplasm in the alloplasmic lines (Martín *et al.*, 2008*b*). PCR was carried out as described by Chung and Staub (2003).

A set of 23 EST markers coded Bawu and K0 (Hagras *et al.*, 2005; Nasuda *et al.*, 2005) that had previously been assigned to chromosomes 1H^ch^ and 6H^ch^ were used to identify the origin of the extra acrocentric chromosome. In addition, two SSRs (Bmac 316 and EBmac 674) (Ramsay *et al.*, 2000), one STS MWG620 (Sayed-Tabatabaei *et al.*, 1998), the *TaFAd* gene (Xu *et al.*, 2008) and the *AK362725* gene (Matsumoto *et al.*, 2011) were also used for the identification of chromosomes.

All amplification products were resolved by agarose gel electrophoresis and visualized with ethidium bromide.

### Transmission of the acrocentric chromosome and fertility scoring

The male and female transmission rates of the acrocentric chromosome were determined cytologically by somatic chromosome counting. To distinguish between male and female transmission, plants carrying the acrocentric chromosome (T700) were crossed with CS both as a male and a female parent, and the transmission rate of the acrocentric chromosome was analysed in the progeny (Supplementary Fig. S1). Selfed progeny of T700 were also investigated.

To compare fertility and morphology of the different lines involving 6H^ch^S, 1H^ch^S, and the acrocentric chromosome under field conditions, the appropriate crosses were carried out to obtain different combinations of these chromosomes in the alloplasmic wheat (T21) background with *H. chilense* cytoplasm. Alloplasmic line T218 was pollinated with T21A1H_1_S to obtain the monosomic addition of 1H^ch^S in the alloplasmic wheat background. Line T593 was pollinated with T21 to obtain the monosomic addition of 6H^ch^S, and with T21A1H_1_S to obtain the monosomic addition of 1H^ch^S and 6H^ch^S. Fertility was scored by counting the number of grains per lateral flower in 20 flowers located in the middle of every spike. Only the first five spikes in every plant were scored.

## Results

### Origin and molecular structure of the acrocentric chromosome

*DArT makers.* In this study, alloplasmic and restored lines were hybridized with an array composed of a total of 4941 DArT clones, most of them derived from *H. chilense* and hexaploid wheat probes. The array was complemented with markers from barley, rye, and triticale. Wheat-*H. chilense* addition lines were also included, allowing the assignment of 1280 *H. chilense* markers to specific chromosomes.

Restored lines T528 (42+ac″) and T700 (42+ac′) gave positive signals in the array with markers assigned to chromosomes 1H^ch^S and 6H^ch^S exclusively, which indicated that the acrocentric chromosome H^ch^ac was formed by only these two chromosome arms of *H. chilense*. From the 1280 DArT markers specific to *H. chilense* chromosomes, 105 and 128 were assigned to the short arms of chromosome 1H^ch^ and 6H^ch^, respectively. All the markers assigned to 1H^ch^S gave a positive signal in the lines carrying the acrocentric chromosome, whereas only 59 markers (46%) assigned to 6H^ch^S gave a positive signal. DArT markers from 1H^ch^S and 6H^ch^S that gave positive signals in the acrocentric chromosome are shown in Supplementary Material (Supplementary Table S1).

**Fig. 1.**
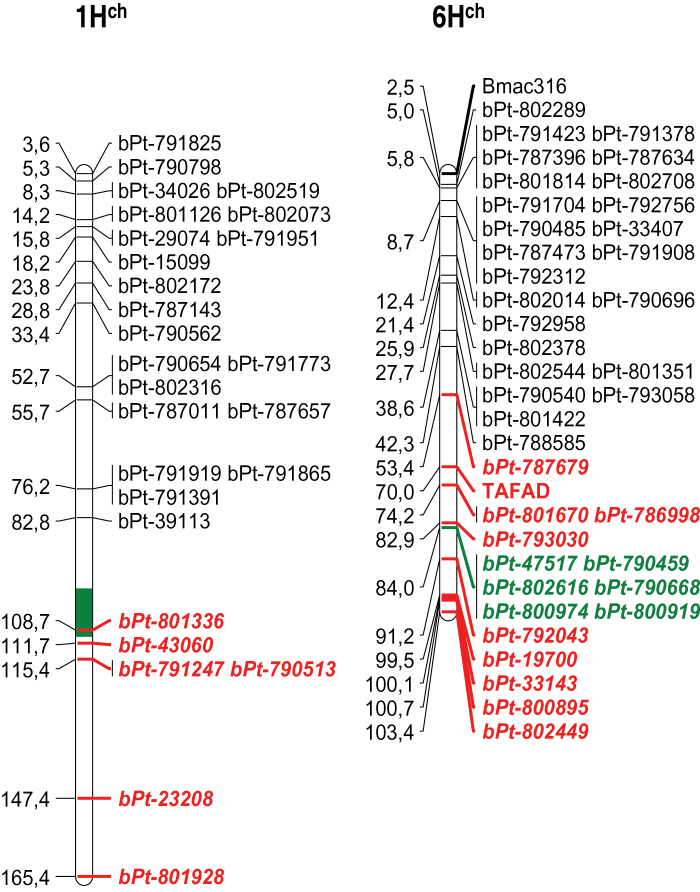
Visualization of markers present in the acrocentric chromosome in the map of *H. chilense* (partial view of chromosomes 1H^ch^ and 6H^ch^). Markers in red were absent in the acrocentric chromosome. The centromeric region, as estimated in previous works, is shown in green. Markers in green are located in the centromeric region.

Four hundred and fifty DArT markers used in this work had already been genetically mapped in a previous *H. chilense* mapping project carried out by our group (Rodríguez-Suarez *et al.*, 2012). Fifty-five of them were mapped to a specific *H. chilense* 1H^ch^S or 6H^ch^S chromosome arm. Two non-DArT markers (Bmac 316 and TAFad) were also mapped (Rodríguez-Suárez and Atienza, 2012). All the markers mapped to chromosome 1H^ch^S were present in the acrocentric chromosome. However, several centromeric markers from 6H^ch^S were absent in the acrocentric chromosome, which suggested that not the whole 6H^ch^S arm was present in the acrocentric chromosome (Fig. 1), but only the distal part of it.

*PCR-based marker.* After DArT marker analysis, a set of chromosome-specific PCR-based markers were used to verify the presence of 1H^ch^S and 6H^ch^S chromosomes in the H^ch^ac chromosome. Of the 28 markers tested, 12 produced an amplification fragment corresponding to *H. chilense*. Five of them were assigned to 1H^ch^S and seven to chromosome 6H^ch^S. The presence or absence of these markers in the acrocentric chromosome is shown in Table 2.

**Table 2.** PCR-based markers used to identify the origin of the acrocentric chromosome and to assign to 1H^ch^S and 6H^ch^S chromosomes

Marker/ gene	Chromosome	1H^ch^	1H^ch^S	6H^ch^	6H^ch^S	H^ch^ ac
Bawu 343	1H^ch^S	+	+	–	–	+
K00856	1H^ch^S	+	+	–	–	+
Bawu 842	1H^ch^S	+	+	–	–	+
K08237	1H^ch^S	+	+	–	–	+
AK362725	1H^ch^S	+	+	–	–	+
K03302	6H^ch^S	–	–	+	+	+
Bmac 316	6H^ch^S	–	–	+	+	+
MWG 620	6H^ch^S	–	–	+	+	+
K01385	6H^ch^S	–	–	+	+	+
EBmac674	6H^ch^S	–	–	+	+	–
Bawu94	6H^ch^S	–	–	+	+	–
TAFad	6H^ch^S	–	–	+	+	–

**Fig. 2.**
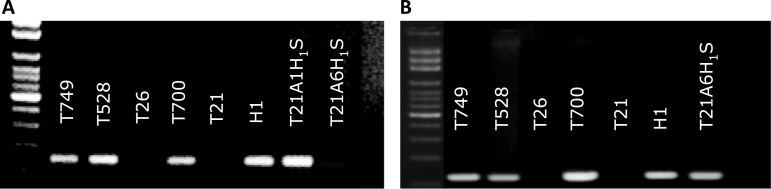
PCR amplification products using (A) primer pairs designed in the AK362725 gene from *H. vulgare* that amplifies specifically the 1H^ch^S chromosome in *H. chilense* and does not produce an amplification product in wheat; (B) SSR marker Bmac 316 that amplifies specifically the 6H^ch^S chromosome in *H. chilense*. T749, T21-*H. chilense* disomic addition of H^ch^ac in H1 cytoplasm; T528, T26-*H. chilense* disomic addition of H^ch^ac in H1 cytoplasm; T26, *T. aestivum* carrying the translocation T1RS·1BL; T700, T21-*H. chilense* monosomic addition of H^ch^ac in H1 cytoplasm; T21, *T. aestivum* cv. Chinese Spring; H1, *H. chilense* accession H1; T21A1H_1_S, T21 ditelosomic addition of 1H^ch^S; and T21A6H_1_S, T21 ditelosomic addition of 6H^ch^S.

All of the markers from 1H^ch^S amplified a product in the acrocentric chromosome. Of the seven markers located on 6H^ch^S, four amplified in H^ch^ac. As observed in Fig. 2, the presence of chromosomes 1H^ch^S and 6H^ch^S was confirmed in the acrocentric chromosome.

### Characterization of the acrocentric chromosome by FISH and meiotic pairing analysis

**Fig. 3.**
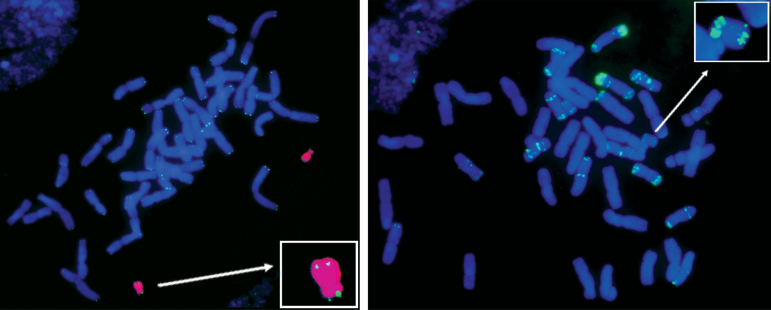
*In situ* hybridization to root-tip metaphase cells from restored line T749. (A) Double FISH signals using *H. chilense* genomic DNA detected with streptavidin–Cy3 (magenta) and a telomere repeat sequence probe detected with FITC (green). Blue DAPI staining shows wheat chromosomes. The acrocentric chromosome H^ch^ac displays magenta colour indicating its pure barley origin. Telomere sequences can be observed at both ends of the H^ch^ac chromosome. (B) FISH signal using the repetitive probe pAs1 detected with FITC. pAs1 shows a characteristic hybridization pattern in *H. chilense* that allows for the identification of the different chromosome arms. The H^ch^ac chromosome shows hybridization sites in both arms.

**Fig. 4.**
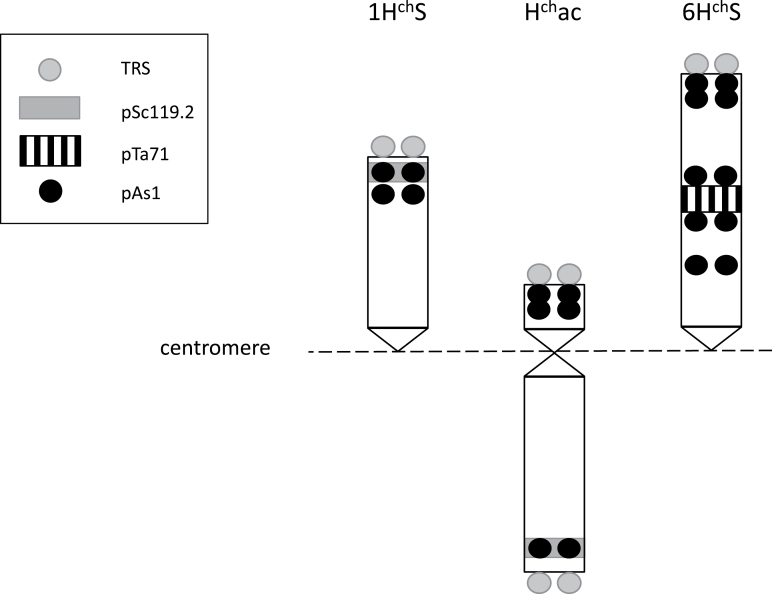
Graphical representation showing the locations of telomere repeat sequences (TRS), pSc119.2, pTa71 rDNA, and pAs1 repetitive probes in 1H^ch^S, 6H^ch^S, and the acrocentric chromosome H^ch^ac.

*FISH.* To show the unique barley origin of the acrocentric chromosome, T749 line (42+ac″) was analysed by GISH using *H. chilense* H1 genomic DNA as probe, labelled with biotin-11-dUTP and detected with streptavidin–Cy3 (magenta). As shown in Fig. 3A, the entire acrocentric chromosome is magenta in colour, indicating its unique *H. chilense* origin. FISH was also carried out using a TRS and the repetitive pAs1 probe, both labelled with digoxigenin-16-dUTP and detected with FITC (green). The TRS probe was used to show that telomeres were present at both ends of the H^ch^ac chromosome arms in spite of the reorganization in this chromosome (Fig. 3A). The pAs1 probe (Fig. 3B) was used because it shows a characteristic hybridization pattern in *H. chilense* that can help the identification of the different chromosome arms. Fig. 3B shows that the H^ch^ac chromosome has hybridization sites in both arms. There is a single hybridization signal at the terminal position of the long arm of H^ch^ac, and two closely spaced signals at the terminal and subterminal positions of the short arm of H^ch^ac. Based on the FISH patterns obtained using the TRS and the pAs1 as probes, together with the patterns using the pTa71 and pSc119.2 as probes described in Martín *et al*. (2010), a graphical representation of the acrocentric chromosome H^ch^ac is shown in Fig. 4. The locations of the different probes agreed with the results obtained with the DArT and the PCR-based markers, confirming the presence of both 1H^ch^S and 6H^ch^S in the acrocentric chromosome.

*Meiotic pairing analysis.* If the acrocentric chromosome is formed by most of 1H^ch^S and part of 6H^ch^S, we should be able to observe some pairing between the acrocentric chromosome and both chromosomes arms. Moreover, the pairing configuration during pachytene, where chromosomes are still quite decondensed, could shed some light on the localization of both 1H^ch^S and 6H^ch^S chromosome segments in the acrocentric chromosome.

**Fig. 5.**
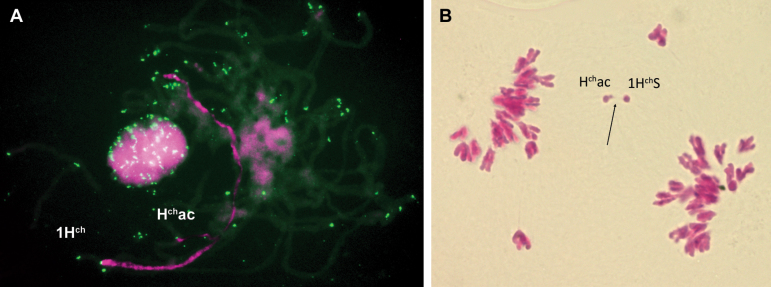
Meiotic pairing analysis of the acrocentric chromosome with 1H^ch^ and 1H^ch^S chromosomes. (A) *In situ* hybridization to a pachytene cell of the double monosomic H^ch^ac-1H^ch^ line. Double FISH signals were observed using *H. chilense* genomic DNA detected with streptavidin–Cy3 (magenta) and a telomere repeat sequence probe detected with FITC (green). The acrocentric chromosome H^ch^ac is perfectly paired with the 1H^ch^ chromosome except for one of its distal parts. (B) Meiotic anaphase I of a plant double monosomic for H^ch^ac and 1H^ch^S stained with carmine. Tension is observed (indicated by an arrow) between the short arm of the acrocentic chromosome and the 1H^ch^S as a result of their pairing.

We first analysed meiotic pairing of the acrocentric chromosome with the whole chromosome 1H^ch^ and the short arm of chromosome 1H^ch^. We used T749 (42+ac″) as female parent and pollinated with T21A1H_1_-1H_1_S (43+t′). In the progeny, two types of combinations were recovered: the double monosomic addition of H^ch^ac and 1H^ch^ and the double monosomic addition of H^ch^ac and 1H^ch^S. Fig. 5A shows a pachytene configuration of the first combination, the double monosomic H^ch^ac-1H^ch^. Telomeres were labelled in green and *H. chilense* DNA in red. It was observed that a large length of the acrocentric chromosome was perfectly paired with 1H^ch^ from one of its ends. Because of the DArT and PCR-based markers, we knew that H^ch^ac contained most, if not the whole, 1H^ch^S chromosome, which together with the FISH results indicated that the terminal region of the acrocentric chromosome was also the terminal region of chromosome 1H^ch^S. Fig. 5B shows an anaphase I of the second combination, the double monosomic addition of H^ch^ac and 1H^ch^S. During metaphase I it was very difficult to assess pairing because all the chromosomes localize at the metaphase plate; however, in anaphase I, when chromosomes were pulled apart, it was occasionally observed that the short arm of the acrocentric chromosome paired with the 1H^ch^S. This supported the hypothesis of 1H^ch^S being part of the short arm of the H^ch^ac.

**Fig. 6.**
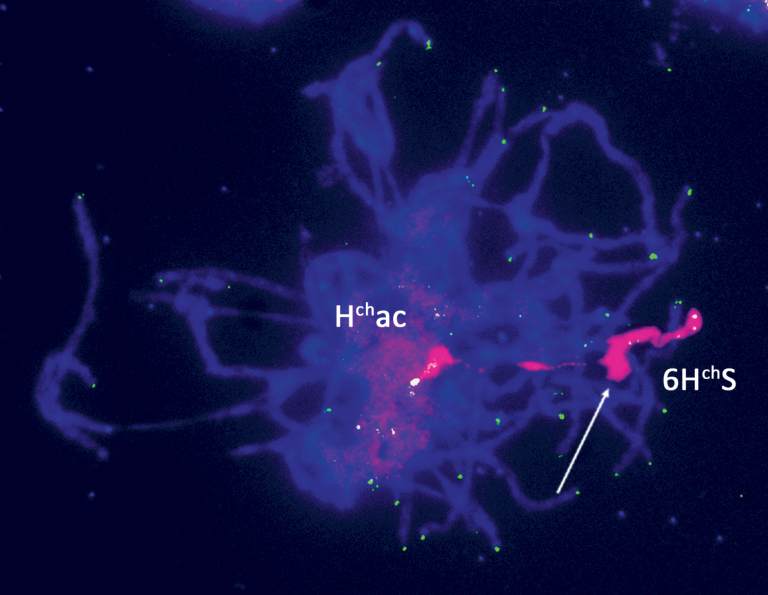
*In situ* hybridization to a pachytene cell from the line double monosomic for H^ch^ac and 6H^ch^S. Double FISH signals were observed using *H. chilense* genomic DNA detected with streptavidin–Cy3 (magenta) and a telomere repeat sequence probe detected with FITC (green). Blue DAPI staining shows wheat chromosomes. The acrocentric chromosome H^ch^ac and the 6H^ch^S chromosome pair along one of their distal parts. The centromeric part of the 6H^ch^S is indicated by an arrow.

Next, to analyse the meiotic pairing of the acrocentric chromosome with chromosome 6H^ch^S, line T749 (42+ac″) was pollinated with T593 (42+t″) to obtain in the progeny a line double monosomic for H^ch^ac and 6H^ch^S (42′+ac′+t′). This line was also analysed at the pachytene stage (Fig. 6). It was observed that the H^ch^ac chromosome paired with the 6H^ch^S along a great part of one of their distal ends. The pericentromeric area of the 6H^ch^S, identified by the lack of telomere sequences, did not pair with the H^ch^ac, suggesting that the centromere and pericentromeric sequences of the H^ch^ac probably came from chromosome 1H^ch^. However, although there was always pairing between H^ch^ac and 6H^ch^S, the pairing configurations were not always the same. Different pairing configurations are shown in Supplementary Fig. S2.

### Transmission of the acrocentric chromosome to the progeny

In the paper of Martín *et al*. (2010), the mono- and disomic addition lines of the acrocentric chromosome were described and characterized; however, no specific data were given on the transmission of this chromosome. In this work, the male and female transmission of the acrocentric chromosome was determined cytologically by somatic chromosome counting. The disomic addition line of H^ch^ac in both T26 and CS background behaved as a perfectly normal line, with all the progeny being identified as disomic additions of H^ch^ac (data not shown). Consistent with this result, when the alloplasmic lines T218 and T236 were pollinated with T749 and T528 respectively, all the progeny inherited one acrocentric chromosome and were fertile (data not shown). As described in the materials and methods, to distinguish between male and female transmission of H^ch^ac, plants carrying one acrocentric chromosome (T700) were selfed, and also crossed with CS both as male and female parents. The progeny was analysed and data is shown in Table 3. It was observed that the transmission of the acrocentric chromosome was better through the female (18.9%) than through the male gametophyte (6.6%), which is normally observed with aneuploid lines.

**Table 3.** Male and female transmission of the acrocentric chromosome when present in monosomic conditionN, chromosome number.

Cross	N=42+2 ac	N=42+1 ac	N=42	Number of plants
T700×T21	0 (0%)	11 (18.9%)	47 (81.0%)	58
T21×T700	0 (0%)	1 (6.6%)	14 (93.3%)	15
Selfing T700	6 (1.80%)	91 (27.7%)	229 (69.8%)	328

### Fertility restoration scoring

**Fig. 7.**
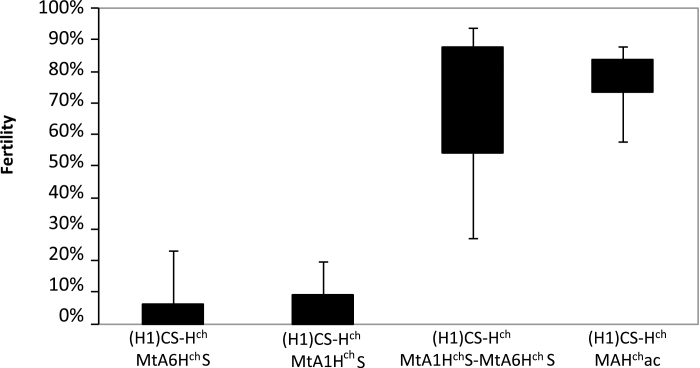
Male fertility restoration of the alloplasmic line Chinese Spring in *H. chilense* cytoplasm, with different chromosome additions: 6H^ch^S, 1H^ch^S, 6H^ch^S+1H^ch^S, and H^ch^ac. (H1)CS-H^ch^ MtA6H^ch^S, CS-*H. chilense* monotelosomic addition of 6H^ch^S; (H1)CS-H^ch^ MtA1H^ch^S, CS-*H. chilense* monotelosomic addition of 1H^ch^S; (H1)CS-H^ch^ MtA1H^ch^S-MtA6H^ch^S, CS-*H. chilense* monotelosomic addition of 1H^ch^S-monotelosomic addition of 6H^ch^S; (H1)CS-H^ch^ MAH^ch^ac, CS-*H. chilense* monosomic addition of H^ch^ac in H1 cytoplasm.

Results obtained in this work demostrate that the acrocentric chromosome is formed by segments of the 1H^ch^S and 6H^ch^S chromosomes of *H. chilense*. Hence, it would be interesting to assess the fertility restoration capacity of these two chromosomes. We evaluated the fertility restoration in alloplasmic lines with the addition of chromosomes 1H^ch^S, 6H^ch^S, and the addition of both chromosome arms. Fig. 7 shows the percentage of restoration in all these cases under the same environmental conditions. The fertility restoration capacity of 1H^ch^S and 6H^ch^S when present in monosomic condition was very low; 3.4 and 5.2%, respectively. However, when both chromosome arms were present (still both in monosomic condition), the fertility restoration capacity increased greatly to 67.4%. These results indicated that some restoration capacity must be present in both 1H^ch^S and 6H^ch^S, but that it was only when the two chromosomes were together in the same plant that the fertility restoration capacity was greatly increased. We studied the fertility restoration of alloplasmic lines with the addition of the acrocentric chromosome (Fig. 7 and Supplementary Table S2). In this case, the fertility capacity restoration was even higher than when both 1H^ch^S and 6H^ch^S were added, reaching 77.6%.

## Discussion

### Origin of the acrocentric chromosome

The acrocentric chromosome described by Martín *et al*. (2010) was suggested to be chromosome 1H^ch^ after a deletion of the distal part of the long arm. However, this was only a hypothesis, as at the time of that study, limited genomic information was available for the wild barley *H. chilense.* Comparative genomics allowed the transfer of wheat and barley molecular markers to *H. chilense* (Hagras *et al.*, 2005; Nasuda *et al.*, 2005), but their density proved to be insufficient for a more detailed study. In 2012, the development of *H. chilense*-derived DArT markers and their use in genetic and physical mapping (Rodríguez-Suárez *et al.*, 2012) allowed us to study the acrocentric chromosome H^ch^ac in greater details. As shown in the results section, the H^ch^ac chromosome gave positive signals with markers assigned to chromosomes 1H^ch^S and 6H^ch^S exclusively, which indicates, unequivocally, that H^ch^ac is formed by these two chromosome arms of *H. chilense*. All the DArT markers assigned to the short arm of chromosome 1H^ch^ gave a positive signal in the lines carrying the acrocentric chromosome, indicating that most, if not all, of 1H^ch^S is probably part of the H^ch^ac chromosome. Only 46% of the DArT markers assigned to chromosome 6H^ch^S gave positive signals in the acrocentric chromosome, which shows that only part of 6H^ch^S is present in H^ch^ac. No positive signal was detected for 1H^ch^L on the acrocentric chromosome. Therefore, the original hypothesis about the presence of a restorer gene on 1H^ch^L seemed to be incorrect.

Some of the DArT markers located in H^ch^ac had already been mapped to the different *H. chilense* chromosomes by Rodríguez-Suárez *et al*. (2012), together with two non-DArT markers, Bmac 316 and TAFad (Rodríguez-Suárez and Atienza, 2012). This allowed us to determine that the centromeric part of 6H^ch^S is not present in the acrocentric chromosome, but only the more distal part (Fig. 1). However, the DArT marker analysis does not define the order of the different chromosome segments in the H^ch^ac chromosome; we only know that most of 1H^ch^S and part of 6H^ch^S are present, but not the order on the H^ch^ac chromosome. To shed some light on this matter, we carried out FISH and meiotic pairing analysis. It was previously shown (Martín *et al.*, 2010) that H^ch^ac did not possess sequences similar to wheat rDNA, so the nucleolar organizer region (NOR) located on 6H^ch^S is not present in H^ch^ac. It was also shown that the pSc119.2 probe hybridized to the long arm of the H^ch^ac chromosome. This data, together with the DArT analysis carried out in this work, indicates that the end of the long arm of H^ch^ac corresponds to the distal part of 1H^ch^S, and that only the distal part of 6H^ch^S beyond the NOR is present in the H^ch^ac chromosome. As expected, FISH using a telomere repeat sequence showed the presence of telomeres at both ends of H^ch^ac. The telomere sequences from the long arm of H^ch^ac must correspond to the ones from 1H^ch^S, but the origin of the telomere sequences present in the short arm of H^ch^ac was not clear. DArT marker analysis showed that the distal part of 6H^ch^S is also part of the acrocentric chromosome, so it seems reasonable to postulate that the telomere sequences present at the end of the short arm of the H^ch^ac chromosome correspond to the telomeres of 6H^ch^S. However, we should also consider the possibility of telomere sequence regeneration *de novo*. Telomeres are necessary for the stability of chromosomes and it is known that telomeres can also form *de novo* at the sites of chromosome breaks in a process termed telomere healing or *de novo* telomere addition (Kramer and Haber, 1993; Friebe *et al.*, 2001; Pennaneach *et al.*, 2006; Zhang *et al.*, 2010). Cytological analyses showed that the acrocentric chromosome pairs with both 1H^ch^S and 6H^ch^S chromosomes, which suggests that the distal parts of the acrocentric chromosomes belong to chromosomes 1H^ch^S and 6H^ch^S. Because both the DArT marker and cytological analyses indicate that only the distal part of chromosome 6H^ch^S is present in the acrocentric chromosome, the simplest explanation for the origin of the acrocentric chromosome would be a fusion between the distal part of 6H^ch^S and the short arm of 1H^ch^, carrying the centromere from the latter. However, the collective cytological data obtained in this work suggests that the origin of H^ch^ac is probably more complex; therefore we propose an alternative hypothesis. Fig. 5A shows that 1H^ch^ and H^ch^ac pair along one of their ends and that the unpaired segment of H^ch^ac is smaller than the paired one. The pairing of chromosome 6H^ch^S with H^ch^ac (Fig. 6) seems to be from the ends as well, leaving the proximal part of 6H^ch^S unpaired. However, whereas the pairing between 1H^ch^S and H^ch^ac was evident and well defined, the pairing of chromosome 6H^ch^S with H^ch^ac was not always so clear (Supplementary Fig. S2). Additionally, anaphase I (Fig. 5B) shows the remaining pairing between the telocentric 1H^ch^S and the short arm of H^ch^ac. This suggests that part of 1H^ch^S is located in the short arm of H^ch^ac. Based on all the results, we propose that H^ch^ac is a `zebra-like′ chromosome (Jiang and Gill, 1993) formed by alternate fragments of chromosomes 6H^ch^S and 1H^ch^S. The short arm of H^ch^ac would be formed by two chromatin segments: the pericentromeric region would be derived from the pericentromeric region of chromosome 1H^ch^S including the centromeric sequences; and the telomeric segment was derived from the telomeric region of 6H^ch^S. The long arm of H^ch^ac would be formed by two chromatin segments as well: the pericentromeric region would include a 6H^ch^S segment, and the rest of the arm would derive from chromosome 1H^ch^S including the telomere. We hypothesize the genesis of H^ch^ac in three steps: first, a centric fusion of 1H^ch^S and 6H^ch^S; second, the deletion of the middle region of 6H^ch^S including the NOR region; and third, a pericentric inversion.

### Restoration of male fertility

The observation of modified additional chromosomes that spontaneously arise when working on interspecific hybridization, and that are associated with CMS and fertility restoration in wheat is not exceptional (Jiang and Gill, 1993; Francki and Langridge, 1994; Zhang *et al.*, 2008). The acrocentric chromosome described in this work is reminiscent of the case of the zebra chromosome first described by Jiang and Gill (1993). The zebra chromosome was isolated from the derivatives of an *Elymus trachycaulus* × *T. aestivum* hybrid and was named `zebra′ because of its striped GISH pattern as a result of multiple translocations involving two non-homologous chromosomes (from *E. tranchycaulus* and *T. aestivum*). The zebra chromosome, the same as the H^ch^ac, is able to restore male fertility in alloplasmic wheat (Zhang *et al.*, 2008). The mechanism of origin of these modified chromosomes could be explained by non-homologous recombination or by multiple translocation events. Zhang *et al.* (2008) suggested that the zebra chromosome originated from non-homologous recombination based mainly on the linear order of the markers of the *Elymus* and wheat segments in the zebra chromosome. In the case of the H^ch^ac chromosome, we suggest that the origin is multiple translocation events (as explained above). However, the exact composition of this chromosome can only be known until the marker order becomes available. Aneuploid changes in chromosome number and the origin of structurally rearranged chromosomes are frequently associated with interspecific hybridization (summarized by Gill, 1991). Whatever the origin of these zebra chromosomes, it seems obvious that these kinds of modified chromosomes are more common than we had originally thought; but it is only in cases where they show some reproductive advantages, such as H^ch^ac restoring male fertility, that they are retained and consequently studied. The significance of this phenomenon should be considered in relation to chromosome evolution and step changes in chromosome number, particularly in polyploid species like wheat, where buffering of the genome owing to polyploidy allows the study of chromosome structure and behaviour over many generations (Jiang and Gill, 1993). An attractive hypothesis is to consider alloplasmic male sterility as a first step leading to unisexual plants, which is one of the mechanisms in the determination of functionally dioecious species. The cellular and molecular mechanisms leading to unisexuality remains poorly understood. Recently, temporal and spatial changes in the pattern of programmed cell death during gametogenesis have been indicated as responsible for male sterility of female flowers (Flores-Renteria *et al.*, 2013). In the msH1 system, flowers initiate as hermaphrodites. As their development progresses, the male reproductive system aborts or collapses (just after microspore formation), whereas the female reproductive system is perfectly functional. In other alloplasmic systems, effects on the male reproductive system are even more marked. An example is pistilloidy (Kihara, 1951; Fukasawa, 1953; Murai *et al.*, 2002), the homeotic transformation of stamens into pistil-like structures. Pistilloidy could be the initiation of a plant with only female flowers, whereas the presence of a fertility restorer could lead to the appearance of the male ones.

Independently of the origin and composition of the acrocentric chromosome, which is very interesting from the point of view of chromosome evolution, the high relevance of this work relies on the ability of this chromosome to restore male fertility. The importance of the group 6 homoeologous chromosomes in the restoration of male fertility has been revealed for different Triticeae species. In wheat, restorer genes for the *T. timopheevii* cytoplasm were located on homoeologous group 6 chromosomes: *Rf4* on 6B, *Rf5* on 6D, and *Rf6* on 6A and 6B (McIntosh *et al*., 2003). The *Rfc3* restorer of rye is localized in the 40% terminal region of chromosome 6R (Curtis and Lukaszewski, 1993). In barley, the *Rfm1* restorer locates on the distal part of chromosome 6HS (Matsui *et al.*, 2001); and recently, in winter triticale with T. timopheevii cytoplasm, the most effective restorer genes were also found on the chromosomes belonging to the homoeologous group 6 (Stojalowski *et al.*, 2013). On the other hand, several restorer genes have also been located on homoeologous group 1 chromosomes in wheat (Robertson and Curtis, 1967; Kuĉera, 1982; Maan *et al.*, 1984; Jiang and Gill, 1994; Ma and Sorrels, 1995; Kojima *et al.*, 1997; Ahmed *et al.*, 2001; Liu *et al.*, 2002; Zhang *et al.*, 2008). In Martín *et al*. (2010), it was mentioned that the addition of 1H^ch^S to wheat did not restore pollen fertility in the alloplasmic wheat; however, we observed that both monosomic addition of chromosome 6H^ch^S or 1H^ch^S occasionally restores fertility to CMS wheat (Fig. 7 and Supplementary Table S2). The fact that fertility restoration of 1H^ch^S is so low and unpredictable (3.4%), suggests that its restoration ability may be highly affected by environmental conditions, which is very frequently observed when working with CMS (Maan *et al.*, 1984; Wilson, 1984; Ma and Sorrells, 1995; Abdel-Ghani *et al.*, 2012). However, when both chromosome arms 6H^ch^S and 1H^ch^S are present in monosomic conditions, the fertility restoration capacity increases to 67.4% (Fig. 7 and Supplementary Table S2). Thus, some restoration capacity is present in both the 1H^ch^S and 6H^ch^S arms, but it is only when the two chromosomes are both present that fertility restoration is greatly increased. The restorer gene located in 6H^ch^S is probably the *Rf*_*6H*_^*ch*^_*S*_ as previously described by Martín *et al*. (2008*a*), whereas a new restorer gene named *Rf*_*1H*_^*ch*^_*S*_ is present on 1H^ch^S. Fertility restoration capacity of the H^ch^ac chromosome is higher than those when both 1H^ch^S and 6H^ch^S are present (77.6% vs. 67.4%). This supports the presence of both *Rf*_*6H*_^*ch*^_*S*_ and *Rf*_*1H*_^*ch*^_*S*_ in the acrocentric chromosome, because the increased fertility can be explained by the presence of both restorers in single gametes. During meiosis, two consecutive nuclear divisions (meiosis I and meiosis II) occur without chromosomal replication in between, leading to the production of four haploid gametes, each containing one of each pair of homologous chromosomes. Therefore the probability of the two *Rf* genes going into the same gamete is much higher if they are present in the same chromosome (H^ch^ac), rather than in two different chromosomes (6H^ch^S and 1H^ch^S).

It is unknown whether both restorer genes, *Rf*_*6H*_^*ch*^_*S*_ and *Rf*_*1H*_^*ch*^_*S*_, are located in the same chromosome arm of H^ch^ac. To shed some light on this question, we screened over 1000 plants trying to recover the short and the long arm of chromosome H^ch^ac by centromeric misdivision at metaphase I. We were not successful, probably because as suggested above, the *Rf*_*6H*_^*ch*^_*S*_ is located on the short arm of H^ch^ac, whereas the *Rf*_*1H*_^*ch*^_*S*_ is on the long arm, and neither of them gives enough advantage to be transmitted alone by the pollen. When the acrocentric chromosome is present in monosomic condition, its transmission to the progeny is better through the female gametophyte than through the male one, which is due to certation; and when H^ch^ac is present in homozygosity, its transmission is 100% to the progeny. Consequently, the restoration capacity of the disomic addition of the acrocentric chromosome line is complete. However, it is not possible to use this system for hybrid seed production if restoration relies on aneuploidy, as the performance of aneuploids will never reach the high and stable performance of an euploid line. In future development of the system, the goal will be to introgress the *Rf* genes present in the H^ch^ac chromosome into euploid wheat.

## Supplementary data

Supplementary data are available at *JXB* online

Table S1. DArT markers assigned to *Hordeum chilense* 1H^ch^S and 6H^ch^S chromosomes.

Table S2. Number of grains per lateral flower in 20 flowers (located in the middle of every spike) and percentage of fertility restoration.

Figure S1. Transmission rate of the acrocentric chromosome when present in monosomic condition.

Figure S2. *In situ* hybridization to pachytene cells from the line double monosomic for H^ch^ac and 6H^ch^S.

Supplementary Data
