# Supplementary material for: Fertility of CMS wheat is restored by two Rf loci located on a recombined acrocentric chromosome
Source: J Exp Bot. 2014 Sep 30;65(22):6667–77. doi: 10.1093/jxb/eru388 (PMC4246193; doi:10.1093/jxb/eru388)
Supplement: Supplementary Data [file supp_65_22_6667__index.html]

Fertility of CMS wheat is restored by two Rf loci located on a recombined acrocentric chromosome — Supplementary Data 

# Fertility of CMS wheat is restored by two *Rf* loci located on a recombined acrocentric chromosome

## Supplementary Data

Data files

**Files in this Data Supplement:**

- Supplementary Data - Supplementary Data
