# Supplementary material for: Fertility of CMS wheat is restored by two Rf loci located on a recombined acrocentric chromosome
Source: J Exp Bot. 2014 Sep 30;65(22):6667–77. doi: 10.1093/jxb/eru388 (PMC4246193; doi:10.1093/jxb/eru388)
Supplement: Supplementary Data [file supp_eru388_NEW_Supplementary_file_combined.pdf]

Fertility of CMS wheat is restored by two *Rf* loci located on a recombined acrocentric chromosome

Almudena Castillo, Sergio G. Atienza, Azahara C. Martín.

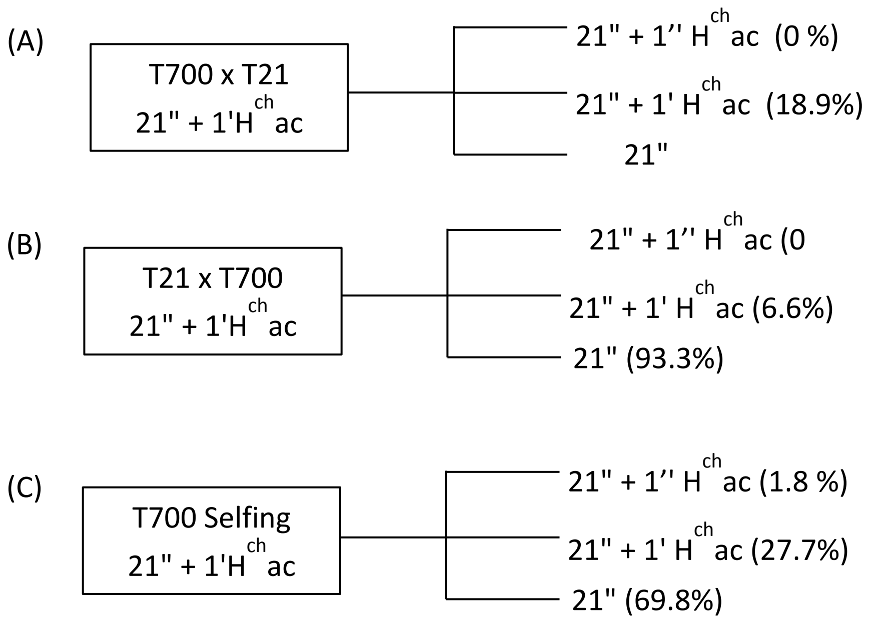

Figure S1. Transmission rate of the acrocentric chromosome when present in monosomic condition. (A) Female transmission of the acrocentric chromosome. (B) Male transmission of the acrocentric chromosome. (C) Selfed progeny of T700.

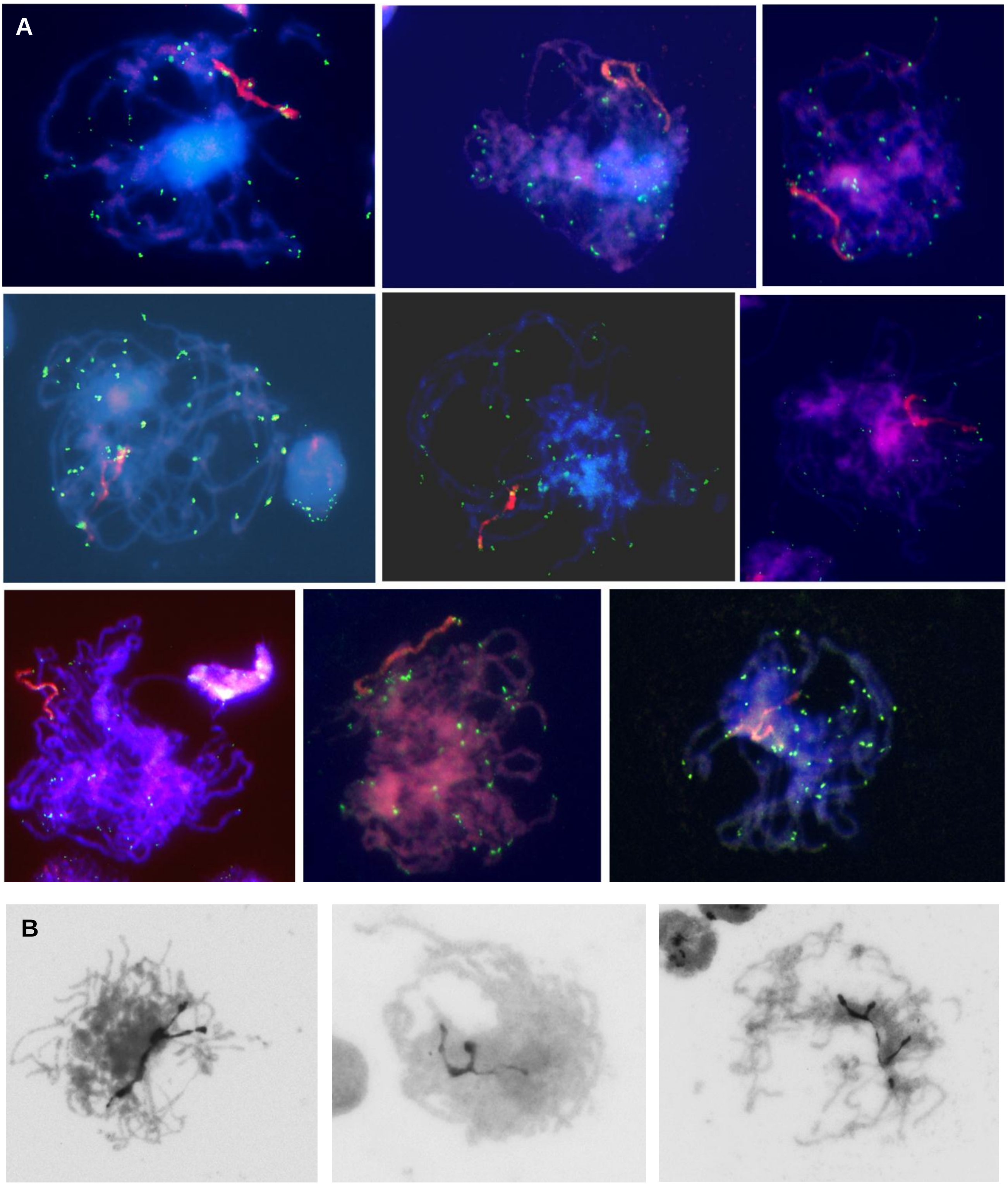

Figure S2. In situ hybridization to pachytene cells from the line double monosomic for H^{ch}ac and $6H^{ch}$S. (A) Double FISH signal using *H. chilense* genomic DNA detected with streptavidin-Cy3 (magenta) and a telomere repeat sequence probe detected with digoxigenin-FITC (green). (B) GISH signal using *H. chilense* genomic DNA detected with streptavidin-Cy3 (dark grey).

Table S2. Number of grains per lateral flower in 10 flowers (located in the middle of every spike) and percentage of fertility restoration. Only the five main spikes were scored in every plant.

Standard abbreviation	1º Spike	2º Spike	3º Spike	4º Spike	5º Spike	% Fertility
(H1)CS-MtA6H^{ch}S-1	0	0	0	0	0	0
(H1)CS-MtA6H^{ch}S-2	0	0	1	0	0	1
(H1)CS-MtA6H^{ch}S-3	2	8	2	0	0	12
(H1)CS-MtA6H^{ch}S-4	0	1	6	0	0	7
(H1)CS-MtA6H^{ch}S-5	0	0	2	0	0	2
(H1)CS-MtA6H^{ch}S-6	0	0	0	0	0	0
(H1)CS-MtA6H^{ch}S-7	0	0	0	0	0	0
(H1)CS-MtA6H^{ch}S-8	2	5	9	7	0	23
(H1)CS-MtA6H^{ch}S-9	1	2	0	2	0	5
(H1)CS-MtA6H^{ch}S-10	0	0	1	0	1	2
(H1)CS-MtA1H^{ch}S-1	0	0	0	0	0	0
(H1)CS-MtA1H^{ch}S-2	0	0	0	0	0	0
(H1)CS-MtA1H^{ch}S-3	0	0	0	0	0	0
(H1)CS-MtA1H^{ch}S-4	0	0	0	0	0	0
(H1)CS-MtA1H^{ch}S-5	3	5	1	2	9	20
(H1)CS-MtA1H^{ch}S-6	0	0	1	1	0	2
(H1)CS-MtA1H^{ch}S-7	0	0	1	0	0	1
(H1)CS-MtA1H^{ch}S-8	0	1	5	0	0	6
(H1)CS-MtA1H^{ch}S-9	0	0	0	0	0	0
(H1)CS-MtA1H^{ch}S-10	0	0	0	0	0	0
(H1)CS-MtA1H^{ch}S-11	0	0	0	0	0	0
(H1)CS-MtA1H^{ch}S-12	0	0	0	0	0	0
(H1)CS-MtA1H^{ch}S-13	4	0	1	0	0	5
(H1)CS-MtA1H^{ch}S-14	0	0	0	0	2	2
(H1)CS-MtA1H^{ch}S-15	0	0	0	0	0	0
(H1)CS-MtA1H^{ch}S-16	0	2	2	1	14	19
(H1)CS-MtA1H^{ch}S MtA6H^{ch}S-1	4	9	7	7	0	27
(H1)CS-MtA1H^{ch}S MtA6H^{ch}S-2	10	7	8	1	2	28
(H1)CS-MtA1H^{ch}S MtA6H^{ch}S-3	11	15	10	15	12	63
(H1)CS-MtA1H^{ch}S MtA6H^{ch}S-4	14	14	15	14	18	75
(H1)CS-MtA1H^{ch}S MtA6H^{ch}S-5	12	19	19	18	20	88
(H1)CS-MtA1H^{ch}S MtA6H^{ch}S-6	16	16	15	16	15	78
(H1)CS-MtA1H^{ch}S MtA6H^{ch}S-7	18	18	20	19	19	94
(H1)CS-MtA1H^{ch}S MtA6H^{ch}S-8	18	17	19	16	16	86
(H1)CS-MAH^{ch}ac-1	16	17	16	20	19	88
(H1)CS-MAH^{ch}ac-2	16	12	16	11	12	67
(H1)CS-MAH^{ch}ac-3	19	19	13	13	16	80
(H1)CS-MAH^{ch}ac-4	18	14	16	17	20	85
(H1)CS-MAH^{ch}ac-5	15	16	17	17	19	84
(H1)CS-MAH^{ch}ac-6	15	15	17	14	15	76
(H1)CS-MAH^{ch}ac-7	10	12	11	12	13	58
